# Individual differences and compliance intentions with COVID-19 restrictions: insights from a lockdown in Melbourne (Australia)

**DOI:** 10.1093/heapro/daac089

**Published:** 2022-07-28

**Authors:** Jaime C Auton, Daniel Sturman

**Affiliations:** School of Psychology, University of Adelaide, Australia; School of Psychology, University of Adelaide, Australia

**Keywords:** COVID-19, individual differences, knowledge, compliance, social distancing

## Abstract

The coronavirus (COVID-19) pandemic has caused an international public health and economic crisis. Despite the COVID-19 vaccine rollout in many countries from late 2020, non-pharmaceutical interventions are still required to minimize the spread of the virus. However, notable variation in voluntary compliance with these interventions has been reported. This study investigated various individual differences associated with intentions to comply with COVID-19 restrictions during a sustained (112 day) lockdown in Melbourne (Australia) in late 2020. Participants (*N* = 363) completed an online survey where they responded to various socio-demographic, health and psychological questions. Participants also responded to a series of vignettes that assessed their intended behaviour in specific situations and their knowledge of the current COVID-19 restrictions. Overall, it was found that greater levels of organization predicted greater intentions to comply with the COVID-19 restrictions, while higher socio-economic status, sociability and anxiety predicted lower compliance intentions. Further, individuals previously diagnosed with COVID-19 reported lower intentions to comply with the COVID-19 restrictions. The strongest predictor of compliance intentions, however, was a greater knowledge of the current restrictions. These findings highlight that public health orders around COVID-19 restrictions should be presented in a clear and uncomplicated manner and should target specific groups to increase compliance.

Since December 2019, the coronavirus disease 2019 (COVID-19) has been an international public health emergency (Bubar *et al.*, 2021; Chang *et al.*, 2020). On 11 March 2020, COVID-19 was declared a global pandemic by the World Health Organization (World Health Organization, 2020, March 11). There have been over 328 million cases and 5.54 million deaths reported worldwide as of January 2022 (Johns Hopkins University Coronavirus Resource Centre, 2022). COVID-19 vaccinations became available in many countries from December 2020, where 7.87 billion COVID-19 vaccine doses have already been administered to individuals in over 50 countries (as of January 2022; Our World in Data, 2020).

Prior to the availability of COVID-19 vaccines, however, non-pharmaceutical interventions (e.g. physical distancing, mandatory mask wearing) were rapidly adopted by countries worldwide as the primary defence against community infection (Beeckman *et al.*, 2020; Coroiu *et al.*, 2020, Fong *et al.*, 2020; Wright *et al.*, 2021). These non-pharmaceutical interventions will still be required alongside the COVID-19 vaccine rollout until herd immunity to the virus is established (Chu *et al.*, 2020; McDermott, 2021). Indeed, one estimate predicts that they may remain necessary until 2022 (Kissler *et al.*, 2020).

There has been variation in the scope, severity and application of these interventions worldwide (McCarthy *et al.*, 2021). In many countries, social (or physical) distancing measures have been commonplace, where individuals are encouraged to maintain a physical distance from each other (Murphy *et al.*, 2020; Pedersen and Favero, 2020). This often includes a limit (or elimination) of public gatherings, closures of schools, universities and businesses. Further, border restrictions have been imposed across and within many countries (Murphy *et al.*, 2020). Other measures, such as increased personal hygiene practices, widespread COVID-19 testing, face mask requirements and mandatory quarantine of confirmed and suspected cases of COVID-19, have also been commonplace.

More restrictive strategies have also been deemed necessary to control the virus in some areas. For example, in certain parts of the world, such as Wuhan (China), Italy and Melbourne (Australia), community-wide containments have been mandated (Carlucci *et al.*, 2020; Coroiu *et al.*, 2020; Smith, 2020). Such lockdowns have required the complete quarantine of the population of an entire region, where individuals are required to stay at home except for essential purposes (Wilder-Smith and Freedman, 2020). While these non-pharmaceutical measures have caused extreme disruption to individuals and communities worldwide, they have been effective in reducing the incidence of COVID-19 (Askitas *et al.*, 2021; Chu *et al.*, 2020; Islam *et al.*, 2020). Without these non-pharmaceutical interventions, it was predicted that COVID-19 would have caused seven billion infections and 40 million deaths globally in 2020 (Walker *et al.*, 2020).

The efficacy of non-pharmaceutical interventions, however, is heavily reliant on individuals’ acceptance and adherence to these rules and restrictions (Carlucci *et al.*, 2020; Hills and Eraso, 2021). Unfortunately, notable variation in voluntary compliance with these interventions has been reported. For example, a recent survey found that 39.8% of American respondents were not complying with all social distancing recommendations (Moore *et al.*, 2020). In Italy, a study of quarantined adults found that constant adherence to various preventative behaviours varied depending on the behaviour. Adherence ranged from 18% (avoidance to touch mouth/eyes) to 92.8% (avoidance of gatherings; Carlucci *et al.*, 2020). A survey of North London residents found that 48.6% intentionally flouted social distancing rules (Hills and Eraso, 2021). In Australia, it has been documented that only 21.2% of those surveyed complied with all COVID-19 restrictions in place at the time of inquiry (Murphy *et al.*, 2020). Compliance with COVID-19 government restrictions and recommendations has not been uniform, with some individuals demonstrating greater compliance than others (McCarthy *et al.*, 2021).

There is a substantial literature on the individual factors associated with compliance with government restrictions during the COVID-19 pandemic, as well as during previous pandemics (e.g. Bish and Michie, 2010; Hills and Eraso, 2021; Webster *et al.*, 2020). It is well established that several demographic (e.g. gender, age), social (e.g. socio-economic status), health (e.g. the greater susceptibility of COVID-19, previous diagnosis of COVID-19) and psychological (e.g. personality, anxiety) factors are associated with the engagement of social distancing behaviours during a pandemic (Hills and Eraso, 2021). While there is clear evidence to suggest that a range of factors are associated with compliance behaviours, consistent associations are not repeatedly found across studies.

While compliance with COVID-19 restrictions varies across a range of factors, knowledge of the current public health requirements (e.g. mandatory mask-wearing in public places) is another individual factor that would likely affect compliance. Logically, the extent of voluntary compliance with government directives would depend upon individuals having a clear understanding of what they are required to do. During the H1N1 pandemic in Victoria (Australia) in 2009, for example, individuals with a greater understanding of the quarantine rules demonstrated higher rates of quarantine compliance (Kavanagh *et al.*, 2011). However, research into the factors that predict voluntary compliance with COVID-19 public health measures has largely focused on knowledge of the COVID-19 virus (e.g. clinical presentation, transmission, control) while overlooking the importance of knowledge and understanding of the current restrictions (Azlan *et al.*, 2020; Honarvar *et al.*, 2020; Wright *et al.*, 2021; Zhong *et al.*, 2020).

Arguably, there would be a significant individual variation in the knowledge and understanding of COVID-19 restrictions at any given time. As virus outbreaks within many countries have been rapid and unpredictable, individuals have been required to adapt to constantly changing government restrictions from sources that vary greatly in accuracy and clarity. However, researchers have largely ignored the possibility that knowledge and understanding of these restrictions can vary between individuals.

Indeed, compliance with COVID-19 restrictions has been frequently assessed by researchers asking individuals to respond to very broad questions, such as ‘*I only leave home for reasons sanctioned by the government’* (Clark *et al.*, 2020, p. 77), ‘*Are you following the recommendations from authorities to prevent spread of Covid-19*?’ (Wright *et al.*, p. 3) or ‘*I strictly followed my state’s preventative measures (e.g. social distancing, wearing a mask) during the COVID-19 outbreak’* (Wang *et al.*, 2021, p. 2). This approach assumes that individuals possess an accurate and complete understanding of the current COVID-19 measures which may have resulted in distorted compliance rates. It is critical to understand whether knowledge of COVID-19 restrictions is positively associated with compliance. This might provide further insights into how the provision of public health information can be curated for maximum efficacy.

## THE CURRENT STUDY

The aim of this study was to investigate the relationship between various demographic, social, health and psychological factors (including knowledge of restrictions) and intentions to adhere to the COVID-19 restrictions. Participants comprised residents from the city of Melbourne (Australia) who were in the final three weeks of Stage 4 lockdown restrictions (Australia’s highest level of restrictions) in September 2020. Participants completed a cross-sectional online survey where they were asked to respond to a series of socio-demographic, health and psychological questions. To assess knowledge of and intentions to adhere to the current COVID-19 restrictions, participants were asked to read and respond to a series of hypothetical scenarios (vignettes) where the protagonists were either adhering to or violating the current restrictions. Intentions to adhere to the current COVID-19 restrictions represented the dependent variable.

The use of vignettes enabled environmental conditions to be standardized across participants, as individuals’ adherence to restrictions was likely to be dependent on situational factors. An individual’s specific situation may have made it easy to adhere to restrictions, while unexpected circumstances (sick child/relative, job demands) may have motivated individuals to violate restrictions. It has also been shown that self-reported intentions of behaviours in a hypothetical scenario predict actual behaviour in real life (e.g. Rossetto *et al.*, 2016). Thus, the use of vignettes provided insight into participants’ current knowledge and intended behaviour in a variety of situations potentially faced during the pandemic.

Based on the previous literature, it was hypothesized that knowledge of restrictions, age, socio-economic status, conscientiousness (diligence and organization) and anxiety would predict greater intentions to comply with the COVID-19 restrictions, while extraversion (sociability) would predict lower intentions to comply with restrictions. Further, it was hypothesized that females and individuals with increased susceptibility to COVID-19 would report greater intentions to comply with the COVID-19 restrictions, whereas individuals previously diagnosed with COVID-19 would report lower intentions to comply with the COVID-19 restrictions.

## METHOD

### Participants

In response to the second wave of COVID-19, residents of the metropolitan city of Melbourne (Australia) entered one of the world’s toughest lockdowns on 7 July 2020 (Smith, 2020). Over a period of 112 days, the city’s five million residents were constrained to strict stay at home orders of varying intensities until 27 October 2020 (Smith, 2020). On 2 August 2020, a state of disaster was declared, and Melbourne residents were moved from Stage 3 restrictions to the more stringent Stage 4 restrictions for six weeks (Tsirtsakis, 2020). During Stage 4 restrictions, residents were subject to a curfew from 8 p.m. until 5 a.m. each day. Outside of these hours, residents were instructed to stay at home except for permitted reasons (i.e. shopping for essential items, exercise [maximum of 1 h per day], medical care, or approved work). Even within these parameters, residents were only permitted to travel within 5 km of their residence and were required to maintain a social distance of 1.5 m from others (Cooper, 2020). Stage 4 restrictions eased in Melbourne from 15 September 2020.

For the purposes of this study, participants were required to be residents of Melbourne over the age of 18 and fluent in English. Recruited through the Qualtrics Market Research Panels, participants comprised 363 residents of Melbourne who completed the survey between 28 August 2020 and 14 September 2020 (the last three weeks of the Stage 4 restrictions).

### Materials

#### Knowledge of and intentions to comply with restrictions

To assess knowledge of and intentions to comply with the Stage 4 restrictions mandated in Melbourne, a series of 15 vignettes were developed. Each vignette consisted of a short-written scenario, where the protagonist/s were either complying with or violating the Stage 4 restrictions (e.g. ‘*Max and Lisa both work in a hospital. Recently they have both been working extremely long hours, and are often too tired to clean when they get home. They have recently hired a cleaner who comes to their home to clean once a fortnight. The cleaner always maintains physical distancing and wears a face covering at all times*.’). Out of the 15 vignettes, the protagonist/s were in violation of the Stage 4 restrictions in 12 of the vignettes and were not violating the restrictions in the remaining three vignettes. Examples of behaviours that were in violation of the Stage 4 restrictions included traveling more than 5 km from a private residence (for a reason not permitted), visiting the private residence of another person or engaging the services of an individual (e.g. cleaner, babysitter) within a private residence.

Participants were instructed that they would be presented with several hypothetical scenarios and were required to assess whether the individual(s) involved were violating the current Stage 4 restrictions in place for metropolitan Melbourne (e.g. ‘*By hiring a cleaner to come to their home, Max and Lisa are violating the current restrictions’* [True/False]). To assess participants level of knowledge of the current restrictions, the number of correct responses was summed to provide a score out of 15.

To assess participants’ intentions to comply with the current restrictions (dependent variable), they were then asked to indicate whether they would perform the same behaviour exhibited by the individual/s in the scenario (e.g. ‘*If I were Max and Lisa, I would get a cleaner to come to my home’*). Responses were made on a 6-point Likert scale from 1 (*Strongly Disagree*) to 6 (*Strongly Agree*). For the 12 vignettes in which the protagonists were violating the restrictions, the scores were reverse coded. The scores across the 15 vignettes were summed to create an ‘Intentions to Comply with COVID-19 Restrictions’ score out of 90. Higher scores on this variable indicated a greater intention to adhere to the Stage 4 restrictions.

#### Demographic factors

Participants were asked to indicate their age (in years) and their identified gender (male, female, other, prefer not to say).

#### Social factors

The Index of Relative Socio-economic Advantage and Disadvantage (IRSAD) was used as a measure of socio-economic status (SES). Calculated by the Australian Bureau of Statistics (ABS, 2016), this index summarizes information about the economic and social conditions of individuals and households within a geographical area, including factors such as household income, educational level and occupation type. This index uses a decile rating to rank suburbs using postcodes from 1 (most disadvantaged) to 10 (most advantaged). Participants were asked to provide their residential postcodes which were then used to allocate participants a decile rating on the IRSAD. This decile rating represented a participant’s SES score.

#### Health factors

To assess participants’ susceptibility or previous diagnosis of the COVID-19 virus, participants were asked to respond to two items. Participants were asked to indicate whether they had an increased susceptibility to COVID-19 (yes/no). Participants were also asked to indicate whether they had been diagnosed with COVID-19 (yes/no).

#### Psychological factors

The personality factors of conscientiousness and extraversion were measured using the HEXACO-60, a short measure of the major dimensions of personality (Ashton and Lee, 2009). Two of the four facets of conscientiousness (organization, diligence) and one of the four facets of extraversion (sociability) were measured in this study. The Organization scale assesses an individual’s tendency to establish order, particularly within physical surroundings (Lee and Ashton, 2009). For organization, participants were asked to respond to two items: ‘*I plan ahead and organize things, to avoid scrambling at the last minute’* and ‘*When working, I sometimes have difficulties due to being disorganized*’ (reverse coded).

The diligence scale assesses an individual’s hardworking and persevering nature. For diligence, participants were asked to respond to two items: ‘*I often push myself very hard when trying to achieve a goal’* and ‘*I do only the minimum amount of work needed to get by’* (reverse coded).

The sociability scale assesses an individual’s preference to enjoy conversation, social interaction and social gatherings. Similarly, sociability was measured using two items: ‘*I prefer jobs that involve active social interaction to those that involve working alone’* and ‘*The first thing that I always do in a new place is to make friends’*. For all items, responses were made on a 5-point Likert scale from 1 (*Disagree*) to 5 (*Agree*). For each facet, the mean of the scores for the two items was calculated, with higher scores denoting higher levels of the facet.

Anxiety was assessed using the affective wellbeing scale (anxiety/contentment; Warr, 1990). Participants were asked to rate the extent to which they had experienced three different responses over the past four weeks (tense, uneasy and worried). Participants responded on a 5-point Likert scale from 1 (*Never*) to 5 (*Always*). Mean scores were calculated (Cronbach’s *α* = 0.91), with higher scores denoting higher feelings of anxiety.

### Procedure

The survey was administered via the Qualtrics online survey platform and was available for the last three weeks of Stage 4 restrictions (28 August 2020—14 September 2020). After reading the information form and providing informed consent, participants responded to a series of demographic, social, health and psychological questions. Participants then read and responded to the social distancing vignettes. Participants took an average of 23 min to complete the survey.

## RESULTS

Ranging in age from 19 to 86 years (*M* = 44.41, SD = 15.73), participants comprised 203 females (55.9%) and 160 males (44.1%). The majority of participants (79.6%) reported living with others (e.g. spouse, children and housemates), with the remaining participants living alone. The majority of participants (75.8%) were working throughout the duration of Stage 4 restrictions for an average of 33.85 hours per week (SD = 18.54). Of those who were working, 234 (85.1%) indicated they were working from home during the Stage 4 restrictions.

Descriptive statistics are displayed in Table 1 and bivariate correlations are displayed in Table 2. Overall, there was a positive correlation between intentions to comply with the COVID-19 restrictions and diligence, organization and knowledge of restrictions (*p* < 0.05) and a negative correlation with sociability and anxiety (*p* < 0.05).

**Table 1:** Descriptive statistics

Variable	*n*	%	Mean	SD.	Min.	Max.
Gender						
Female	203	55.9				
Male	160	44.1				
Age			44.41	15.73	19	86
Socio-economic status			7.32	2.50	1	10
Previous COVID-19 diagnosis						
Yes	9	2.5				
No	354	97.5				
COVID-19 susceptibility						
Yes	97	26.7				
No	266	73.3				
Conscientiousness (Diligence)			3.65	0.85	1	5
Conscientiousness (Organisation)			3.72	0.84	1.5	5
Extraversion (sociability)			3.44	0.93	1	5
Anxiety			2.38	1.10	1	5
Knowledge of restrictions (/15)			9.42	2.40	3	14
Intentions to comply with restrictions (/90)			43.76	9.91	12	61

**Table 2:** Bivariate correlations

Variable	1	2	3	4	5	6	7	8
1. Age	1							
2. Socio-economic status	0.05	1						
3. Conscientiousness (diligence)	0.12*	−0.00	1					
4. Conscientiousness (organisation)	0.25**	−0.03	0.39**	1				
5. Extraversion (sociability)	−0.04	−0.02	−0.04	−0.00	1			
6. Anxiety	−0.31**	−0.06	−0.07	−0.30**	0.07	1		
7. Knowledge of restrictions	0.10	0.07	0.03	0.16**	−0.01	−.11*	1	
8. Intentions to comply with restrictions	0.09	−0.06	0.13*	0.25**	−0.22**	−0.27**	.043**	1

* <0.05 (2-tailed). ** <0.01 (2-tailed).

To examine which demographic, social, health and psychological factors were associated with individuals’ intentions to adhere to the COVID-19 restrictions, a multiple linear regression (backward elimination) was conducted. Data screening and assumption testing indicated the assumptions of independence, normality, constant variance and linearity were met. All independent variables (age, gender, SES, COVID-19 diagnosis, COVID-19 susceptibility, diligence, organization, sociability, anxiety and knowledge) were entered into the first regression model, with intentions to adhere to the COVID-19 restrictions as the dependent variable. Model 2 excluded the variable COVID-19 susceptibility and Model 3 further excluded the variable Diligence. Model 4 showed the best fit, further excluding the variable Age. Table 3 presents the results from Model 1 (full model) and Model 4 (final model) of the regression analysis.

**Table 3:** Multiple linear regression (backward elimination): predictors of intentions to comply with Melbourne stage 4 COVID-19 restrictions (*N* = 363)

Variables	Model 1 (full model)			Model 4 (final model)			
	*b* (SE)	*β*	*p*	*b* (SE)	*β*	*p*	VIF
Gender (0 = female)	−1.38 (.88)	−0.07	0.119	−1.65 (0.86)	−0.08	0.054	1.01
Age	−0.03 (.03)	−0.05	0.273	−	−	-	-
Socio-economic status	−0.40 (.17)	−1.00*	0.023	−0.40 (.17)	−0.10*	0.021	1.02
COVID-19 Diagnosis (0 = no)	−14.29 (2.86)	−0.22***	<.001	−14.14 (2.80)	−0.22***	<0.001	1.07
COVID-19 susceptibility (0 = no)	0.07 (.99)	0.00	0.947	–	–	–	–
Conscientiousness (diligence)	0.51 (.56)	0.04	0.364	–	–	–	–
Conscientiousness (organization)	0.98 (.59)	0.08	0.096	1.08 (0.54)	0.09*	0.045	1.15
Extraversion (sociability)	−2.05 (.46)	−0.19***	<0.001	−2.05 (0.46)	−0.19***	<0.001	1.01
Anxiety	−1.57 (.42)	−0.17***	<0.001	−1.44 (0.41)	−0.16***	<0.001	1.13
Knowledge of restrictions	1.82 (0.18)	0.44***	<0.001	1.80 (0.18)	0.44***	<0.001	1.07
(constant)	37.18 (3.87)***		<0.001	37.24 (3.50)***		<0.001	
*R* ^2^	0.357			0.354			
Adjusted *R*^2^	0.339			0.341			
*R* ^2^ change	0.357			−0.002			
*F* change	19.58***		<0.001	1.08		0.299	

*Note.* Bolded coefficients represent those that are significant at *p* < 0.001 or less. *p < 0.05; **p < 0.01; ***p < 0.001.

Overall, the independent variables contained in Model 4 (final model) accounted for 35.4% of the variability in the dependent variable (intentions to adhere to the COVID-19 restrictions), *F*(7,355) = 27.79, *p* < 0.001. For the demographic factors, it was found that females demonstrated greater intentions to adhere to the restrictions, compared to their male counterparts (*β* = − 0.08, *p* = 0.054). However, this gender difference was only bordering on statistical significance. There was no association found between age and intentions to adhere to the COVID-19 restrictions. There was a statistically significant negative association between SES and intentions to comply with the COVID-19 restrictions (*β* = −0.10, *p* = 0.021). This suggests that those who had a higher SES possessed lower intentions to comply with the restrictions. This association was in the opposite direction than hypothesized.

With reference to the health factors, there was no statistically significant association found between individual susceptibility to COVID-19 and intentions to adhere to the restrictions. However, there was a negative association found between COVID-19 diagnosis and intentions to adhere to restrictions (*β* = −0.22, *p* < 0.001) as hypothesized. These results suggest that individuals who had never been diagnosed with COVID-19 possessed greater intentions to adhere to the COVID-19 restrictions, compared to those who had contracted the virus.

With reference to the psychological factors, diligence had no association with intentions to adhere to restrictions. As hypothesized, there was a statistically significant positive association between organization and intentions to adhere (*β* = 0.09, *p* = 0.045). Individuals who had higher levels of organization demonstrated greater intentions to comply with the restrictions compared to their less organized counterparts. There was a statistically significant negative association between sociability and intentions to comply with restrictions (*β* = −0.19, *p* < 0.001). As hypothesized, individuals who had higher levels of sociability had lower intentions to comply with the restrictions compared to those who had lower levels of sociability. Contrary to the direction hypothesized, anxiety was negatively associated with intentions to comply with COVID-19 restrictions (*β* = −0.16, *p* < 0.001). Individuals who had higher levels of anxiety had lower intentions to comply with the restrictions compared to their less anxious counterparts.

Finally, those who had greater knowledge of the COVID-19 restrictions had greater intentions to comply with the COVID-19 restrictions, compared to their less knowledgeable counterparts (*β* = 0.44, *p* < 0.001). Comparing the beta weights across the independent variables, knowledge was the strongest predictor of intentions to comply with the COVID-19 restrictions.

## DISCUSSION

The overall aim of this study was to investigate the association between various demographic, social, health and psychological factors and intentions to comply with Stage 4 COVID-19 restrictions during a localized lockdown in Melbourne (Australia). Of particular interest was the relationship between knowledge of the COVID-19 restrictions and compliance intentions. Overall, it was found that greater knowledge of restrictions and levels of organization predicted greater intentions to comply with the COVID-19 restrictions, while higher socio-economic status, sociability and anxiety predicted lower intentions to comply with restrictions. Further, individuals diagnosed with COVID-19 reported lower intentions to comply with the COVID-19 restrictions. While not quite reaching statistical significance, females did demonstrate greater intentions to adhere to the restrictions, compared to their male counterparts. The remaining individual differences (age, COVID-19 susceptibility and diligence) were not associated with intentions to adhere to the COVID-19 restrictions.

Previous research investigating factors predicting compliance to restrictions during COVID-19 and previous pandemics has been somewhat mixed, with some factors predicting compliance in some contexts but not in others. Unsurprisingly, the results from this study are consistent with some previous findings, but not with others. With reference to demographic factors, for example, older individuals have been found to be more compliant with COVID-19 restrictions, compared to younger individuals (e.g. Brouard *et al.*, 2020; Coroiu *et al.*, 2020; Murphy *et al.*, 2020; Pedersen and Favero, 2020; Wright and Fancourt, 2020). However, the findings from this study, as well as others (e.g. Clark *et al.*, 2020), demonstrate that age is not always related to compliance intentions and behaviours. Gender has been consistently associated with rule adherence in previous studies, where females are more likely to engage in protective behaviours (e.g. social distancing) during the COVID-19 pandemic, compared to their male counterparts (Brouard *et al.*, 2020; Clark *et al.*, 2020; Coroiu *et al.*, 2020; Honarvar *et al.*, 2020; Murphy *et al.*, 2020; Nivette *et al.*, 2021; Pedersen and Favero, 2020; Uddin *et al.* 2021). While not reaching statistical significance, the findings from this study support the existing literature where females, compared to males, were found to have greater compliance intentions.

Supporting previous findings that report an association between higher socio-economic status and lower compliance with COVID-19 restrictions (Nivette *et al.*, 2021; Pedersen and Favero, 2020; Wright and Fancourt, 2020), this study found that higher socio-economic status predicted lower intentions to comply with COVID-19 restrictions. With reference to health factors, this study did not find an association between perceived COVID-19 susceptibility and compliance intentions. While there is some evidence to suggest that vulnerability to COVID-19 has little association with health behaviours (Clark *et al.*, 2020), there are several studies that contrast the findings of this study to highlight that those individuals with a greater perceived susceptibility to the COVID-19 virus are more likely to report higher levels of adherence (Hills and Eraso, 2021; Murphy *et al.*, 2020; Yıldırım *et al.*, 2021). While the available evidence suggests that a previous COVID-19 diagnosis is not associated with adherence to public health directives (Hills and Eraso, 2021), this study found that individuals who had been diagnosed with COVID-19 actually reported lower intentions to comply with COVID-19 restrictions.

The association between some psychological factors, such as personality, and compliance behaviours in past research has been mixed. Conscientiousness has been found to be positively associated with adherence to public health recommendations, whereas extraversion has been negatively associated with adherence to recommendations (Brouard *et al.* 2020). However, other studies have failed to find an association between personality factors and rule following behaviours (Clark *et al.*, 2020). In this study, not all facets of personality were associated with compliance intentions. Specifically, extraversion (sociability) was negatively associated with compliance intentions, where only one facet of conscientiousness (organization) was positively associated with compliance intentions. Finally, this study found that those with higher anxiety demonstrated lower intentions to comply with restrictions, which is consistent with the past research that shows greater levels of psychological distress, fear or anxiety are associated with greater compliance with public health recommendations (e.g. Brouard *et al.*, 2020; Coroiu *et al.*, 2020).

Overall, previous empirical findings in conjunction with the current findings indicate that the importance of various individual factors in predicting compliance behaviours and intentions differs between studies. A potential explanation for these discrepancies could be the range of dependent variables used to measure compliance behaviours and intentions within the literature. For example, some studies have measured how frequently participants have engaged in restricted behaviours during a specified time frame (e.g. Coroiu *et al.*, 2020; Honarvar *et al.*, 2020; Hills and Eraso, 2021; Murphy *et al.*, 2020), whereas others have measured the extent to which participants agree they have been following all government rules and protective behaviours (e.g. Clark *et al.*, 2020; Nivette *et al.*, 2021; Wright and Fancourt, 2020) or even a blended measure of both compliance intentions and actual behaviour (e.g. Beeckman *et al.*, 2020).

While these self-report measures of compliance behaviours used in previous studies are potentially subject to recall bias and socially desirable responses, the ability to which individuals can adhere to COVID-19 restrictions might also differ due to situational circumstances (e.g. job demands) rather than specific demographic, health, social and psychological factors. Rather than relying on a self-report measure of compliance to COVID-19 restrictions, a strength and point of difference of the current study was the use of vignettes that enabled a standardized measure of compliance intentions that were independent of individuals’ situational factors. As such, the lack of consistent associations between various factors and compliance intentions and behaviours in previous literature may be, in part, a function of how compliance has been operationalized.

Another explanation for these inconsistent findings is that individual predictors of compliance are likely to be dependent upon situational and contextual factors, such as country of residence or type of lockdown restrictions. Specifically, this study examined associations between individual factors and compliance intentions during a prolonged period of lockdown under very strict conditions. It may be the case that these individual factors are not as relevant or generalizable to other situations where lockdowns are much shorter and/or less restrictive. These results do highlight, however, certain demographic groups that are less likely to engage in sustained social distancing efforts. These groups could potentially benefit from targeted messaging about the importance and necessity of continued compliance with COVID-19 restrictions.

While this study highlighted several individual factors associated with compliance intentions, knowledge of the COVID-19 restrictions was found to be the strongest predictor of compliance intentions. This finding can help guide the transmission of public health information around COVID-19 restrictions during future outbreaks. For example, future public health promotions should focus on presenting COVID-19 restrictions in a simplified manner to ensure that the restrictions are easy to understand and easy to remember. Further, all platforms that are regularly used as an information source (e.g., social media) should contain accurate and updated information. This will ensure widespread dissemination of information and help reduce mixed messaging, which may lead to non-compliance (Wang *et al.*, 2021). This approach toward clear and accurate public health messaging is of current importance for Australian residents. As Australia began to relax its national and international border restrictions from November 2021, social distancing measures are still required to manage the spread of the virus for the foreseeable future and hence, accurate knowledge of these measures is required.

The key limitations of this study and areas for future research should be noted. First, the survey conducted was cross sectional in nature. This means that the causal nature of the associations between variables cannot be established. For example, it may be that those individuals who intend to comply with the COVID-19 restrictions are more motivated to seek out information and knowledge about the restrictions, rather than knowledge driving compliance intentions. Using a longitudinal design, future research could examine the extent to which knowledge of the COVID-19 restrictions predicts intentions to comply with the restrictions. While assessing compliance intentions was advantageous in this cross-sectional survey as it allowed participants to respond to standardized situations, a longitudinal design would also enable an examination of compliance intentions and actual compliance with social distancing behaviours.

While it is impractical to do so, it must be acknowledged that this study did not include an exhaustive and complete list of individual variables that might be associated with compliance intentions. A range of other variables have been found to be associated with actual compliance and compliance intentions that were not included in this study (such as trust in government, family support, highest qualification gained and political affiliation; Hills and Eraso, 2021). Future research could examine whether these variables are important in predicting compliance behaviours during a prolonged lockdown. Future research could also examine whether different individual factors predict knowledge of the COVID-19 restrictions.

## CONCLUSION

This study found that socio-economic status, previous COVID-19 diagnosis, personality (organization, sociability) and anxiety were all associated with compliance intentions amongst participants within a localized lockdown in Melbourne, with the strongest predictor being knowledge of the COVID-19 restrictions. These outcomes suggest that targeted health messaging for non-compliant groups and clear transmission of updated COVID-19 restrictions might be valuable approaches to increase community adherence.
